# Expenses of Motor Upkeep.—II

**Published:** 1909-04-10

**Authors:** 


### Motoring Notes.

### EXPENSES OF MOTOR UPKEEP.—II.

In my last article under this heading (page 671, March 27, 1909) I drew attention to the most important points having a direct bearing on the expense of keeping a motor-car, but there still remain one or two matters worthy of consideration in this connection. Some owners keep the same car for years, whilst there are others who are not satisfied with anything unless it be of the latest pattern and design, and who consequently make it a rule to have a new car every year. There are not, I imagine, many medical men who fall into the latter category, yet there are many who make frequent changes. I think I may say that the principal item of expense in regard to keeping a car is the depreciation in selling value, and the owner who frequently purchases a new car must be prepared to lose in this way proportionately more than the owner who, having a good car, keeps it for several years. Cars of the highest reputation depreciate very much less than others, because their qualities are known and there is a regular demand for them second-hand. Cars of second-rate quality, although their first cost may be considerably less, cause much greater losses, as they have to be resold at a very low figure, owing to the small demand for them.

The selling value does not always represent the actual depreciation from a user's point of view, and, provided the right car is purchased at the beginning and a change is not made, the depreciation is very small indeed. It has been found by actual experience that a really first-class single-cylinder car costing under £250 will, if properly looked after and maintained, easily run over 100,000 miles. Now taking the average motorist's annual mileage at 5,000, this car is capable of 20 years' service, and therefore the annual depreciation will be only £12 10s., or at the rate of 5 per cent. It is, of course, unlikely that anyone would care to run the same car for such a lengthy period as 20 years; yet it could be done, and I give the illustration with a view of proving that, provided a first-class car is purchased and used for several years, depreciation is by no means the bogey that some people would lead us to expect.

There is one final point bearing on expense of upkeep I would mention. It is wise to have the engine and car looked over occasionally, so that parts may be renewed as they become worn. Neglect of this causes excessive wear, and extra expenses eventually accrue. Frequently the neglect to replace a part at the cost of a few shillings will result in the wear of other parts which will cost as many pounds to make good.

Although from time to time letters have appeared in the motoring journals from owners giving their expenses for car upkeep for a year, and sometimes for a number of years, these communications refer to cars of different make, size, and power, and are useless to base any estimate on as to the annual cost of running a car suitable for the "man of moderate means," which classification, I regret to say, includes the doctor nowadays. Last year, however, the De Dion-Bouton Company, who have consistently catered for the medical profession, and whose small car is, I am sure, the most popular with the profession, having some 508 doctors on their records as owners of De Dion cars, wrote to several asking for particulars of the actual expenses they have incurred in running their cars. The replies they received, being the actual experiences under the normal conditions of doctors' use, are most valuable when summarised both to the actual and prospective medical motorist.

In reproducing them in these columns. I must say that they include all expenses except driver or cleaner, motor-house, and depreciation, which vary according to circumstances. To prevent confusion and increase the value of the analysis as a guide, the single-cylinder only is included in the summary.

*Petrol.*—The reports show a total mileage of 97,379, and the cost for petrol £263 3s. 6d. Averaging the price paid for petrol at 14d. a gallon, it means a consumption of 4,511 gallons at the rate of 21.617 miles per gallon.

*Lubricating Oil and Grease.*—In the reports where the expenses for oil and grease are quoted, the mileage amounts to 93,229 and the cost £48 4s. 2d., —0.12d. a mile.

*Mechanical Repairs and Replacements.*—In the reports where these items are separated from others the mileage totals 97,379 and the cost £124 16s. 3d., —0.3d. a mile.

*Tyre Repairs and Replacements.*—In the reports where these items are stated the mileage totals 92,879 and the costs £210 15s. 6d.—0.54d. a mile.

*Sundry Running Costs.*—Running costs not included under the above headings amount to £28 7s. for a mileage of 97,379—0.069d. a mile.

Based on the average of the above analysis, the cost of running a single-cylinder De Dion-Bouton car 5,000 miles, which is about the distance travelled in one year by a doctor, would be:—

| | | | |
|---|---|---|---|
| Petrol ... ... ... ... ... | £13 | 10 | 2 |
| Lubricating oil and grease ... ... | 2 | 11 | 8 |
| Mechanical repairs, etc. ... ... | 6 | 4 | 2 |
| Tyre repairs and replacements ... | 11 | 6 | 11 |
| Sundries ... ... ... ... | 1 | 9 | 1 |
| Carriage and driving licences ... ... | 2 | 7 | 0 |
| Insurance against all risks (up to £300) ... ... ... ... | 8 | 5 | 0 |
| | £45 | 14 | 0 |

This sum may be safely taken as a guide as to the expense of running a small car for a year. Possibly, however, the expenses during the first year should prove considerably less, since if tyres of a large section were fitted the cost under this heading should be reduced to a few shillings for repairs of punctures. Some garage proprietors whom I know are prepared, when they sell a car of this type, to find petrol, oil, tyres, keep the car in running order, and insure against all risks for the sum of £35 the first year, £45 the second and third, and £55 the fourth and fifth. When the trade is prepared to do this, the cost of keeping a small car cannot be considered excessive.

